# Microbial biotechnology addressing the plastic waste disaster

**DOI:** 10.1111/1751-7915.12775

**Published:** 2017-07-17

**Authors:** Tanja Narancic, Kevin E. O'Connor

**Affiliations:** ^1^ UCD Earth Institute and School of Biomolecular and Biomedical Science University College Dublin Belfield Dublin 4 Ireland; ^2^ BEACON ‐ Bioeconomy Research Centre University College Dublin Belfield Dublin 4 Ireland

## Abstract

Oceans are a major source of biodiversity, they provide livelihood, and regulate the global ecosystem by absorbing heat and CO_2_. However, they are highly polluted with plastic waste. We are discussing here microbial biotechnology advances with the view to improve the start and the end of life of biodegradable polymers, which could contribute to the sustainable use of marine and coastal ecosystems (UN Sustainability development goal 14).

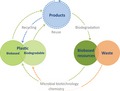

In 2015, global plastic production reached 322 million tonnes, with China accounting for 27.8% of world's plastic production, followed by the EU and USA contributing 18.5% each to world plastic production (Europe, 2016). The packaging segment makes up 39.9% of the plastic market. These mainly single‐use, disposable products greatly contribute to the convenience of modern life. However, their low recycling rates means we are producing large volumes of waste and the plastic waste fraction of municipal solid waste (MSW) is increasing. In the EU and the USA, 31% and 53%, respectively, of MSW including plastics are landfilled (EC, 2015; EPA, 2015).

While plastics pose many problems for terrestrial environments, plastic waste is also a major pollutant in the world's Oceans, resulting in death of wildlife (Rochman *et al*., 2013a,b; Wilcox *et al*., 2015). While the evidence for the presence of plastic in virtually all marine niche is overwhelming (Carson *et al*., 2011; Claessens *et al*., 2011; Frère *et al*., 2017; Kanhai *et al*., 2017), it is unknown how much plastic exactly is in the oceans and in what form (Cressey, 2016). Up to 12.7 million tonnes of plastic waste, generated in 2010 by 192 costal countries, ended up in the ocean (Jambeck *et al*., 2015). Data on microplastic concentrations and toxicity seem to be open for debate (Cressey, 2016). However, there is evidence that environmentally relevant concentrations of microplastic negatively affect sea life (Sussarellu *et al*., 2016). Over three billion people depend on marine and coastal biodiversity (UN, 2015): oceans provide food, medicines and other biobased products (EC, 2012). Furthermore, oceans are buffering the impacts of global warming by absorbing approximately 30% of the CO_2_ in the atmosphere (UN, 2015). Therefore, careful management of this essential global resource is of great importance for sustainability and it is recognized as one of the goals of the 2030 Agenda for sustainable development (UN, 2015).

An effective way to improve resource efficiency and reduce the environmental impact of plastics is the prevention of waste. The EU and EPA strategies for waste management include prevention, reuse, recycling, other recovery and disposal as the last resource (EC, 2013b; EPA, 2014). However, it is realistic to assume that post‐consumer plastics will end up in unmanaged environments. To reduce or prevent the negative impacts of post‐consumer plastic waste, society could replace conventional plastic materials with biodegradable counterparts. Biodegradable plastics can contribute to a more sustainable society using renewable resources, contribute to the reduction in CO_2_ emissions during production and offer new end‐of‐life management options that have a lower or no negative impact on the environment (EC, 2013a; Bioplastics, 2016). Biodegradable polymers are degradable in nature and include polylactic acid (PLA), thermoplastic starch (TPS), polyhydroxyalkanoate (PHA), polycaprolactone (PCL) and poly(butylene adipate‐co‐terephthalate) (PBAT). While PLA, TPS and PHA are also biobased, PCL and PBAT are fossil based. Thus, the origin of the polymer does not necessarily affect its end‐of‐life fate. Indeed, the biobased or natural origins of a polymer do not mean it is biodegradable. For example, technologies are emerging that can make polyethylene (PE), polyethylene terephthalate (PET) from biobased resources, but they are not biodegradable and thus their origin will not address the end‐of‐life pollution challenges.

PLA made up 5.1% of global bioplastic production in 2016, while PHA was represented by 1.6% of 4.16 million tonnes of globally produced bioplastic (Bioplastics, E., 2016). Even though PHAs have desirable properties such as elasticity, hydrophobicity, low oxygen permeability and biodegradability, they have not fully penetrated the plastic market (total production estimated at single figure thousands of tonne) due to uncompetitive pricing compared to fossil based plastics.

PLA and PHA are of a microbial origin, biobased and biodegradable and therefore address both the start and end of the plastic life cycle. PHAs are entirely a product of microbial metabolism, while PLA is produced through a combination of fermentation (to produce lactic acid) and chemistry to convert the lactic acid or lactide to PLA. PHAs are a family of intracellular polyesters that include polymers with very different physical properties (from highly crystalline and brittle to amorphous liquids), which opens up opportunities for different applications from packaging to medicine (Chen, 2009).

Researchers have attempted to address both the cost of production and waste management using different types of waste, including plastic waste, as a cheap feedstock for PHA production, for example polystyrene (Goff *et al*., 2007), polyethylene terephthalate (PET) (Kenny *et al*., 2012), waste glycerol (Cavalheiro *et al*., 2009), animal‐based waste streams (Titz *et al*., 2012), syngas obtained by municipal solid waste (MSW) pyrolysis (Revelles *et al*., 2016) as well as using low cost biomass (Cerrone *et al*., 2015; Walsh *et al*., 2015).

Of particular interest to the emerging circular economy is the upcycling of plastic waste into biodegradable plastic (Goff *et al*., 2007; Kenny *et al*., 2008, 2012; Wierckx *et al*., 2015). While conventional recycling technologies are available, there are several limitations, including cost and relatively low quality of the recycled polymers. Employing the microbial cell factory to convert plastic waste into high value product provides an alternative to conventional recycling. Due to extreme recalcitrance of plastics to microbial degradation, this biotechnological process currently employs pyrolysis to produce oils, which are subsequently fed to bacteria (Goff *et al*., 2007; Kenny *et al*., 2012). However, microbial hydrolases capable of modifying or degrading plastics have emerged recently as a potential technology for plastic biodepolymerization (Wei and Zimmermann, 2017) allowing for a completely biological recycling of plastics. These enzymes could be tailored using the synthetic biology toolbox and then integrated into a microbial chassis to design a custom microbial platform capable of converting plastic into biodegradable counterparts in a single cell (www.p4sb.eu).

The concept of a microbial platform relates also to the concept of minimal cell (Nikel *et al*., 2014). Desirable features of a minimized cell are efficient cell reproduction with minimal genetic drift, efficient control of transcription and translation, and predictable metabolic interactions. The deletion of the flagellar machinery, four prophages, two transposons and three components of DNA restriction‐modification systems in a PHA producer *Pseudomonas putida* KT2440 yielded a minimized cell which achieved higher specific growth rates and higher biomass, tolerated endogenous oxidative stress better, acquired and replicated exogenous DNA, and survived better in stationary phase (Martínez‐García *et al*., 2014). In addition, the bacterial morphology could be engineered to allow larger space for storage of PHA and convenient downstream processing (Jiang and Chen, 2016). Even though several targets have been identified for bacterial morphology modification, this concept of morphology engineering is still novel and requires development.

While PLA sales are growing year on year, the production cost can be reduced further. The microbial conversion of cheap substrates (Zhang and Vadlani, 2013; Muller *et al*., 2017) and waste (Panesar and Kaur, 2015; Pleissner *et al*., 2017) into lactic acid has been investigated. In parallel with efforts to improve the chemical synthesis of PLA (Dusselier *et al*., 2015), enzymatic polymerization of lactide is underway (Lassalle and Ferreira, 2008; Jeon *et al*., 2013). Furthermore, a synthetic pathway containing propionate‐CoA transferase from *Clostridium propionicum* and *Pseudomonas* sp. MBEL 6‐19 PHA polymerase was introduced into *Escherichia coli*, which allowed conversion of glucose into lactyl‐CoA and its polymerization into homopolymer PLA or into a heteropolymer poly(3‐hydroxybutyrate‐co‐lactate) (Yang *et al*., 2010). However, the production of the homopolymer was very poor giving only 0.5% of the cell dry weight as PLA, but it is a promising first step.

Given the additional challenge to improve the thermal and mechanical properties of new biobased and biodegradable polymers, copolymers have been generated (Yang *et al*., 2013). For example, a novel lactic acid containing terpolyester poly(lactate‐co‐3‐hydroxybutyrate‐co‐3‐hydroxypropionate) was produced by a recombinant *E. coli* (Ren *et al*., 2017). The engineered pathway contains 3‐hydroxypropionyl‐CoA synthesis pathway from glycerol, 3‐hydroxybutyryl‐CoA and lactyl‐CoA (LA‐CoA) pathways from glucose and an engineered PHA polymerase from *P. stutzeri* (Ren *et al*., 2017). Metabolically engineered *E. coli* is capable of converting renewable and sustainable resources, glucose and glycerol into the novel terpolyester. Furthermore, just by varying glucose‐to‐glycerol ratio composition of monomers in the terpolyester could be adjusted, which opens up the possibility to tailor the polymer properties.

The creation of composites of biopolymers can generate new materials with improved properties due to synergistic and additive benefits of the combination of polymers (Zhang *et al*., 1996; Broz *et al*., 2003; Yu *et al*., 2006). The compatibilization of polymers remains a major challenge, but nanotechnology is being studied to address this (Dufresne *et al*., 2013).

The advances in microbial biotechnology are creating exciting possibilities to design novel pathways to known biodegradable polymers, but also pathways to novel biodegradable polymers, which address the start and end of life of materials, for the benefit of consumers and the environment. While EU and US EPA place prevention at the top of waste management solutions, certain applications, that is fishing, would inevitably lead to plastic products reaching the ocean. Replacing conventional plastic with biodegradable counterparts should therefore be included into a wider concept of plastic waste management (Fig. 1).

**Figure 1:**
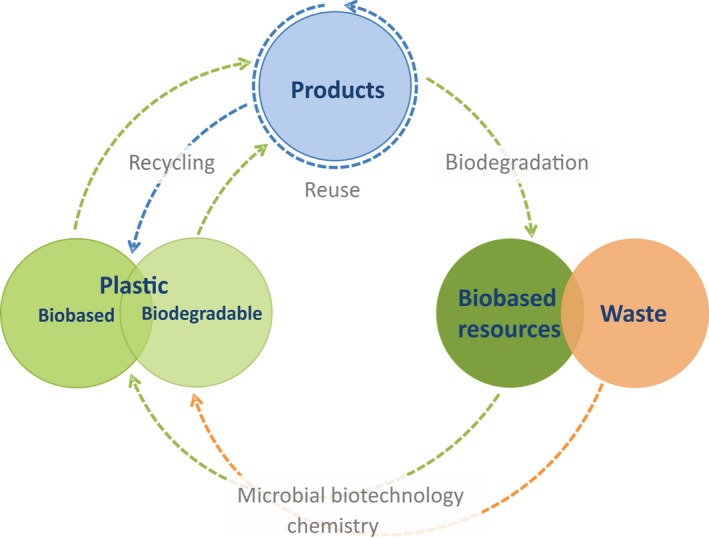
An overview of possibilities created by implementation of biodegradable plastic. Biobased resources and/or waste are used as a feeding stock for the production of plastic, which can be biobased, biodegradable or both. The products made from plastic can be reused, recycled and in the case of biodegradable plastic, that is polyhydroxyalkanoate (PHA), polylactic acid (PLA), thermoplastic starch (TPS) biodegraded to provide new feed stocks for the microbial and/or chemical conversion into plastic, therefore closing the cycle. For applications that would inevitably lead to plastic products reaching the environment, implementation of biodegradable plastic could be used to reduce and prevent the accumulation of plastic waste.

## Conflict of interest

None declared.

## References

[mbt212775-bib-0001] Bioplastics, E. (2016) Driving the evolution of plastics.

[mbt212775-bib-0002] Broz, M.E. , VanderHart, D.L. , and Washburn, N.R. (2003) Structure and mechanical properties of poly(d, l‐lactic acid)/poly(ε‐caprolactone) blends. Biomaterials 24: 4181–4190. 12853248 10.1016/s0142-9612(03)00314-4

[mbt212775-bib-0003] Carson, H.S. , Colbert, S.L. , Kaylor, M.J. , and McDermid, K.J. (2011) Small plastic debris changes water movement and heat transfer through beach sediments. Mar Pollut Bull 62: 1708–1713. 21700298 10.1016/j.marpolbul.2011.05.032

[mbt212775-bib-0004] Cavalheiro, J.M.B.T. , de Almeida, M.C.M.D. , Grandfils, C. , and da Fonseca, M.M.R. (2009) Poly(3‐hydroxybutyrate) production by *Cupriavidus necator* using waste glycerol. Process Biochem 44: 509–515. 10.1016/j.biortech.2012.01.176 22382294

[mbt212775-bib-0005] Cerrone, F. , Davis, R. , Kenny, S.T. , Woods, T. , O'Donovan, A. , Gupta, V.K. , *et al* (2015) Use of a mannitol rich ensiled grass press juice (EGPJ) as a sole carbon source for polyhydroxyalkanoates (PHAs) production through high cell density cultivation. Biores Technol 191: 45–52. 10.1016/j.biortech.2015.04.128 25978856

[mbt212775-bib-0006] Chen, G.‐Q. (2009) A microbial polyhydroxyalkanoates (PHA) based bio‐ and materials industry. Chem Soc Rev 38: 2434–2446. 19623359 10.1039/b812677c

[mbt212775-bib-0007] Claessens, M. , Meester, S.D. , Landuyt, L.V. , Clerck, K.D. , and Janssen, C.R. (2011) Occurrence and distribution of microplastics in marine sediments along the Belgian coast. Mar Pollut Bull 62: 2199–2204. 21802098 10.1016/j.marpolbul.2011.06.030

[mbt212775-bib-0008] Cressey, D. (2016) The plastic ocean. Nature 536: 263–265. 27535517 10.1038/536263a

[mbt212775-bib-0009] Dufresne, A. , Thomas, S. and Pothan, L.A. (2013) Biopolymer Nanocomposites: Processing, Properties, and Applications. John Wiley & Sons, Inc.: Hoboken, NJ, USA.

[mbt212775-bib-0010] Dusselier, M. , Van Wouwe, P. , Dewaele, A. , Jacobs, P.A. , and Sels, B.F. (2015) Green chemistry. Shape‐selective zeolite catalysis for bioplastics production. Science 349: 78–80. 26138977 10.1126/science.aaa7169

[mbt212775-bib-0011] European Commission (2012) Blue Growth: opportunities for marine and maritime sustainable growth.

[mbt212775-bib-0012] European Commission (2013a) GREEN PAPER: On a European Strategy on Plastic Waste in the Environment.

[mbt212775-bib-0013] European Commission (2013b) The Seventh Environment Action Programme to 2020 – ‘Living well, within limits of our planet’.

[mbt212775-bib-0014] European Commission (2015) Directive of the European Parliament and of The Council amending Directive 2008/98/EC on waste.

[mbt212775-bib-0015] Frère, L. , Paul‐Pont, I. , Rinnert, E. , Petton, S. , Jaffré, J. , Bihannic, I. , *et al* (2017) Influence of environmental and anthropogenic factors on the composition, concentration and spatial distribution of microplastics: a case study of the Bay of Brest (Brittany, France). Environ Pollut 225: 211–222. 28371735 10.1016/j.envpol.2017.03.023

[mbt212775-bib-0016] Goff, M. , Ward, P.G. , and O'Connor, K.E. (2007) Improvement of the conversion of polystyrene to polyhydroxyalkanoate through the manipulation of the microbial aspect of the process: a nitrogen feeding strategy for bacterial cells in a stirred tank reactor. J Biotechnol 132: 283–286. 17559958 10.1016/j.jbiotec.2007.03.016

[mbt212775-bib-0017] Jambeck, J.R. , Geyer, R. , Wilcox, C. , Siegler, T.R. , Perryman, M. , Andrady, A. , *et al* (2015) Marine pollution. Plastic waste inputs from land into the ocean. Science 347: 768–771. 25678662 10.1126/science.1260352

[mbt212775-bib-0018] Jeon, B.W. , Lee, J. , Kim, H.S. , Cho, D.H. , Lee, H. , Chang, R. , and Kim, Y.H. (2013) Lipase‐catalyzed enantioselective synthesis of (R, R)‐lactide from alkyl lactate to produce PDLA (poly D‐lactic acid) and stereocomplex PLA (poly lactic acid). J Biotechnol 168: 201–207. 23845270 10.1016/j.jbiotec.2013.06.021

[mbt212775-bib-0019] Jiang, X.‐R. , and Chen, G.‐Q. (2016) Morphology engineering of bacteria for bio‐production. Biotechnol Adv 34: 435–440. 26707986 10.1016/j.biotechadv.2015.12.007

[mbt212775-bib-0020] Kanhai, L.D.K. , Officer, R. , Lyashevska, O. , Thompson, R.C. , and O'Connor, I. (2017) Microplastic abundance, distribution and composition along a latitudinal gradient in the Atlantic Ocean. Mar Pollut Bull 115: 307–314. 28007381 10.1016/j.marpolbul.2016.12.025

[mbt212775-bib-0021] Kenny, S.T. , Runic, J.N. , Kaminsky, W. , Woods, T. , Babu, R.P. , Keely, C.M. , *et al* (2008) Up‐cycling of PET (Polyethylene Terephthalate) to the biodegradable plastic PHA (Polyhydroxyalkanoate). Environ Sci Technol 42: 7696–7701. 18983095 10.1021/es801010e

[mbt212775-bib-0022] Kenny, S. , Runic, J.N. , Kaminsky, W. , Woods, T. , Babu, R. and Oconnor, K.E. (2012) Development of a bioprocess to convert PET derived terephthalic acid and biodiesel derived glycerol to medium chain length polyhydroxyalkanoate. Appl Microbiol Biotechnol 95: 623–633. 22581066 10.1007/s00253-012-4058-4

[mbt212775-bib-0023] Lassalle, V.L. , and Ferreira, M.L. (2008) Lipase‐catalyzed synthesis of polylactic acid: an overview of the experimental aspects. J Chem Technol Biotechnol 83: 1493–1502.

[mbt212775-bib-0024] Martínez‐García, E. , Nikel, P.I. , Aparicio, T. and Lorenzo, V. (2014) Pseudomonas 2.0: genetic upgrading of *P. putida* KT2440 as an enhanced host for heterologous gene expression. Microb Cell Fact 13: 159–174. 25384394 10.1186/s12934-014-0159-3 PMC4230525

[mbt212775-bib-0025] Muller, G. , Kalyani, D.C. , and Horn, S.J. (2017) LPMOs in cellulase mixtures affect fermentation strategies for lactic acid production from lignocellulosic biomass. Biotechnol Bioeng 114: 552–559. 27596285 10.1002/bit.26091

[mbt212775-bib-0026] Nikel, P.I. , Martinez‐Garcia, E. , and de Lorenzo, V. (2014) Biotechnological domestication of pseudomonads using synthetic biology. Nat Rev Microbiol 12: 368–379. 24736795 10.1038/nrmicro3253

[mbt212775-bib-0027] Panesar, P.S. , and Kaur, S. (2015) Bioutilisation of agro‐industrial waste for lactic acid production. Int J Food Sci Technol 50: 2143–2151.

[mbt212775-bib-0028] Plastics Europe (2016) Plastics ‐ the facts 2016.

[mbt212775-bib-0029] Pleissner, D. , Demichelis, F. , Mariano, S. , Fiore, S. , Navarro Gutiérrez, I.M. , Schneider, R. , and Venus, J. (2017) Direct production of lactic acid based on simultaneous saccharification and fermentation of mixed restaurant food waste. J Clean Prod 143: 615–623.

[mbt212775-bib-0030] Ren, Y. , Meng, D. , Wu, L. , Chen, J. , Wu, Q. , and Chen, G.‐Q. (2017) Microbial synthesis of a novel terpolyester P(LA‐co‐3HB‐co‐3HP) from low‐cost substrates. Microb Biotechnol 10: 371–380. 27860284 10.1111/1751-7915.12453 PMC5328817

[mbt212775-bib-0031] Revelles, O. , Beneroso, D. , Menéndez, J.A. , Arenillas, A. , García, J.L. and Prieto, M.A. (2016) Syngas obtained by microwave pyrolysis of household wastes as feedstock for polyhydroxyalkanoate production in *Rhodospirillum rubrum* . Microb Biotechnol [Epub ahead of print]. doi: 10.1111/1751‐7915.12411 10.1111/1751-7915.12411 PMC5658609 27677746

[mbt212775-bib-0032] Rochman, C.M. , Browne, M.A. , Halpern, B.S. , Hentschel, B.T. , Hoh, E. , Karapanagioti, H.K. , *et al* (2013a) Classify plastic waste as hazardous. Nature 494: 169–171. 23407523 10.1038/494169a

[mbt212775-bib-0033] Rochman, C.M. , Hoh, E. , Kurobe, T. and Teh, S.J. (2013b) Ingested plastic transfers hazardous chemicals to fish and induces hepatic stress. Sci Rep‐Uk 3: 3263–3270. 10.1038/srep03263 PMC3836290 24263561

[mbt212775-bib-0034] Sussarellu, R. , Suquet, M. , Thomas, Y. , Lambert, C. , Fabioux, C. , Pernet, M.E. , *et al* (2016) Oyster reproduction is affected by exposure to polystyrene microplastics. Proc Natl Acad Sci USA 113: 2430–2435. 26831072 10.1073/pnas.1519019113 PMC4780615

[mbt212775-bib-0035] http://www.p4sb.eu From plastic waste to plastic value using Pseudomonas putida synthetic biology.

[mbt212775-bib-0036] Titz, M. , Kettl, K.‐H. , Shahzad, K. , Koller, M. , Schnitzer, H. , and Narodoslawsky, M. (2012) Process optimization for efficient biomediated PHA production from animal‐based waste streams. Clean Technol Environ Policy 14: 495–503.

[mbt212775-bib-0037] UN (2015) Sustainable development goals.

[mbt212775-bib-0038] USA EPA (2014) Fiscal Year 2014–2018 EPA Strategic Plan.

[mbt212775-bib-0039] USA EPA (2015) Advancing Sustainable Materials Management: 2013 Fact Sheet.

[mbt212775-bib-0040] Walsh, M. , O'Connor, K. , Babu, R. , Woods, T. , and Kenny, S. (2015) Plant oils and products of their hydrolysis as substrates for polyhydroxyalkanoate synthesis. Chem Biochem Eng Q 29: 123–133.

[mbt212775-bib-0041] Wei, R. and Zimmermann, W. (2017) Microbial enzymes for the recycling of recalcitrant petroleum‐based plastics: how far are we? Microb Biotechnol [Epub ahead of print] doi: 10.1111/1751‐7915.12714. 10.1111/1751-7915.12710 PMC5658625 28371373

[mbt212775-bib-0042] Wierckx, N. , Prieto, M.A. , Pomposiello, P. , de Lorenzo, V. , O'Connor, K. , and Blank, L.M. (2015) Plastic waste as a novel substrate for industrial biotechnology. Microb Biotechnol 8: 900–903. 26482561 10.1111/1751-7915.12312 PMC4621443

[mbt212775-bib-0043] Wilcox, C. , Van Sebille, E. , and Hardesty, B.D. (2015) Threat of plastic pollution to seabirds is global, pervasive, and increasing. Proc Natl Acad Sci USA 112: 11899–11904. 26324886 10.1073/pnas.1502108112 PMC4586823

[mbt212775-bib-0044] Yang, T.H. , Kim, T.W. , Kang, H.O. , Lee, S.‐H. , Lee, E.J. , Lim, S.‐C. , *et al* (2010) Biosynthesis of polylactic acid and its copolymers using evolved propionate CoA transferase and PHA synthase. Biotechnol Bioeng 105: 150–160. 19937726 10.1002/bit.22547

[mbt212775-bib-0045] Yang, J.E. , Choi, S.Y. , Shin, J.H. , Park, S.J. , and Lee, S.Y. (2013) Microbial production of lactate‐containing polyesters. Microb Biotechnol 6: 621–636. 23718266 10.1111/1751-7915.12066 PMC3815930

[mbt212775-bib-0046] Yu, L. , Dean, K. , and Li, L. (2006) Polymer blends and composites from renewable resources. Prog Polym Sci 31: 576–602.

[mbt212775-bib-0047] Zhang, Y. , and Vadlani, P.V. (2013) d‐Lactic acid biosynthesis from biomass‐derived sugars via *Lactobacillus delbrueckii* fermentation. Bioprocess Biosyst Eng 36: 1897–1904. 23670636 10.1007/s00449-013-0965-8

[mbt212775-bib-0048] Zhang, L. , Xiong, C. , and Deng, X. (1996) Miscibility, crystallization and morphology of poly(β‐hydroxybutyrate)/poly(d, l‐lactide) blends. Polymer 37: 235–241.

